# Synthesis and Anticancer Cytotoxicity of Azaaurones Overcoming Multidrug Resistance

**DOI:** 10.3390/molecules25030764

**Published:** 2020-02-10

**Authors:** Szilárd Tóth, Áron Szepesi, Viet-Khoa Tran-Nguyen, Balázs Sarkadi, Katalin Német, Pierre Falson, Attilio Di Pietro, Gergely Szakács, Ahcène Boumendjel

**Affiliations:** 1 Institute of Enzymology, Research Centre for Natural Sciences, Hungarian Academy of Sciences, 1117 Budapest, Hungary; toth.szilard.enzim@ttk.mta.hu (S.T.); aron.szepesi@outlook.hu (Á.S.); balazs.sarkadi@ttk.mta.hu (B.S.); knemet@gmail.com (K.N.); szakacs.gergely@ttk.mta.hu (G.S.); 2 Département de Pharmacochimie Moléculaire (DPM), UMR 5063, Univ. Grenoble Alpes, 38041 Grenoble, France; khoatnv1993@gmail.com or; 3 Creative Cell Ltd., 1119 Budapest, Hungary; 4 Drug Resistance & Membrane Proteins group, Molecular Microbiology and Structural Biochemistry Laboratory (CNRS UMR 5086), University of Lyon, IBCP, 7 Passage du Vercors, 69367 Lyon, France; pierre.falson@ibcp.fr (P.F.); a.dipietro@ibcp.fr (A.D.P.); 5 Institute of Cancer Research, Medical University of Vienna, Borschkegasse 8A, A-1090 Vienna, Austria

**Keywords:** aurone, azaaurone, multidrug resistance, P-gp, overexpression, anticancer, cytotoxicity

## Abstract

The resistance of tumors against anticancer drugs is a major impediment for chemotherapy. Tumors often develop multidrug resistance as a result of the cellular efflux of chemotherapeutic agents by ABC transporters such as P-glycoprotein (ABCB1/P-gp), Multidrug Resistance Protein 1 (ABCC1/MRP1), or Breast Cancer Resistance Protein (ABCG2/BCRP). By screening a chemolibrary comprising 140 compounds, we identified a set of naturally occurring aurones inducing higher cytotoxicity against P-gp-overexpressing multidrug-resistant (MDR) cells versus sensitive (parental, non-P-gp-overexpressing) cells. Follow-up studies conducted with the P-gp inhibitor tariquidar indicated that the MDR-selective toxicity of azaaurones is not mediated by P-gp. Azaaurone analogs possessing pronounced effects were then designed and synthesized. The knowledge gained from structure–activity relationships will pave the way for the design of a new class of anticancer drugs selectively targeting multidrug-resistant cancer cells.

## 1. Introduction

Multidrug resistance constitutes one of the major hurdles to anticancer chemotherapy. One of the well-described mechanisms via which cancer cells become resistant to structurally different chemotherapeutic agents is the overexpression of ATPase efflux pumps belonging to the ABC family of transporters that extrude anticancer drugs from the cells [1,2,3]. Therefore, to ensure efficiency in chemotherapy, higher doses of these medications have to be administered, which, on the other hand, would inevitably lead to severe side effects. In order to counteract multidrug resistance (MDR), one of the established strategies is to develop inhibitors of ABC proteins involved in drug efflux, thus optimizing therapeutic effects of anticancer agents [4]. However, the clinical relevance of such inhibitors remains questionable despite the existence of compounds with potent in vitro activity [2]. Alternatively, recent reports revealed several compounds selectively killing cells overexpressing ATPase efflux pumps, such as P-glycoprotein (P-gp/MDR1) and Multidrug resistance associated protein 1 (MRP1/ABC1) [5,6,7,8,9,10]. These molecules were shown to target MDR cancer cells, offering an emerging strategy for anticancer agent development [11,12].

**Figure 1:**
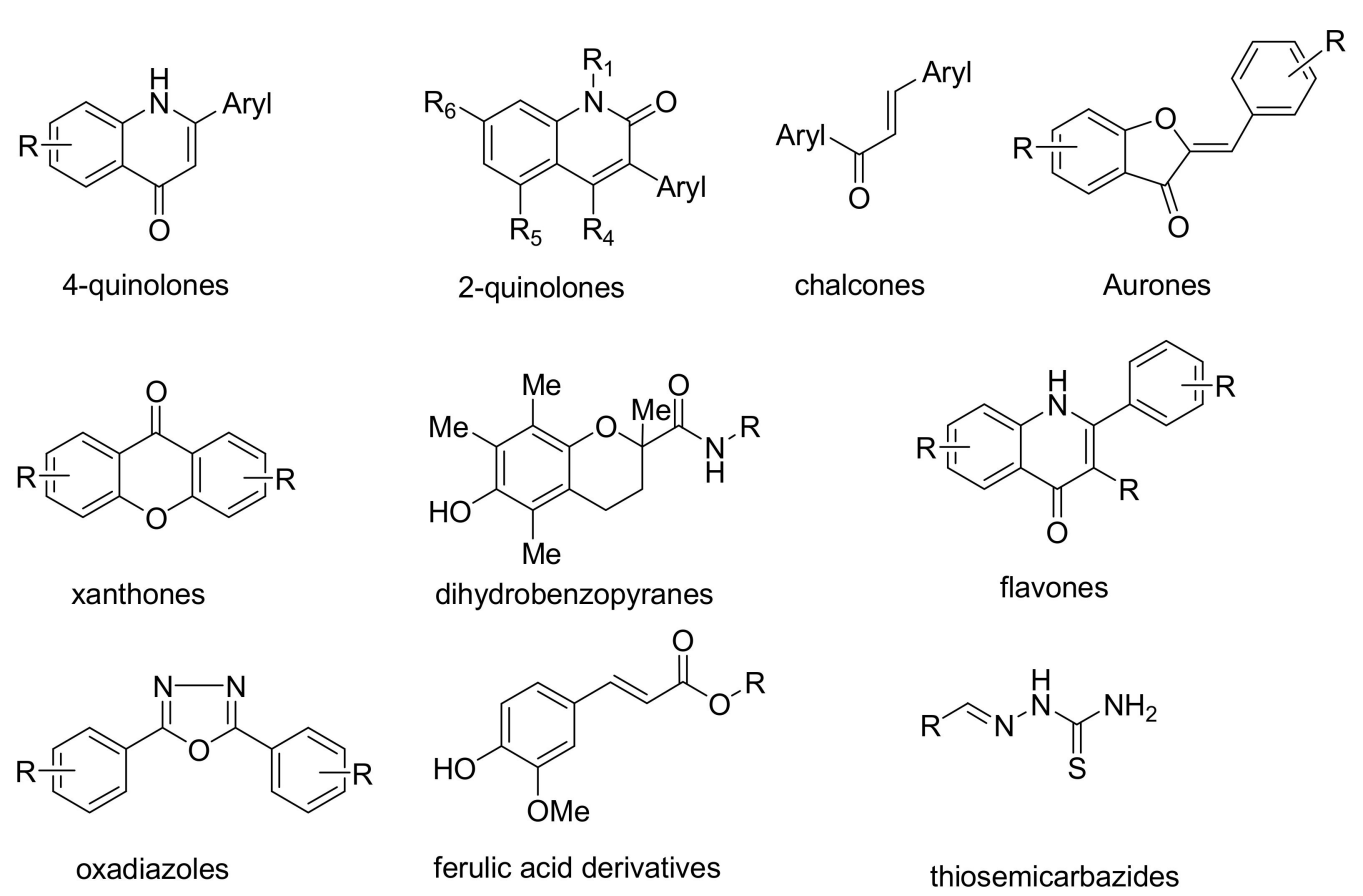
Scaffolds of the derivatives (library of 140 compounds) tested in the primary screen (at 10 and 100 µM).

In this study, we report the result of a cytotoxicity screen performed on a pair of drug-sensitive and multidrug-resistant cancer cell lines. We screened a library of 140 compounds consisting of flavonoidic derivatives and thiosemicarbazones (Figure 1, full structures are provided in Supplementary Materials, Table S1). Flavonoidic derivatives have been widely studied as candidates for cancer treatment and prevention, with various naturally occurring substances reported to be effective against resistant tumors [13,14]. On the other hand, thiosemicarbazones were included since several compounds belonging to this class have been reported to possess increased toxicity against otherwise drug-resistant cells [15,16,17,18]. Primary screening revealed that a subclass of aurones was more toxic to the MDR cell line. Among potential structural analogs of aurones, we found that azaaurones were particularly interesting. Herein, we focused our efforts on the investigation of azaaurones that target the collateral sensitivity of MDR cells. The identified structure–activity relationships will pave the way for the design of more effective compounds with this therapeutic profile.

## 2. Results and Discussion

Primary screening using 140 compounds derived from the scaffolds shown in Figure 1 (Supplementary Materials, Table S1) was conducted using two uterine sarcoma cell lines. Multidrug-resistant MES-SA/Dx5 cells were established from parental MES-SA cells by continuous selection in doxorubicin [19]. The MDR phenotype of MES-SA/Dx5 cells is conveyed by the overexpression of the efflux pump P-glycoprotein. MES-SA/Dx5 cells were cultured in doxorubicin (500 nM), and P-gp expression was regularly checked with the calcein assay (Supplementary Materials, Figure S1) [20].

Compounds were assigned into 3 main categories according to their toxicity against the two cell lines. Of the 140 tested compounds, 8% were toxic in both cell lines (higher than 50% growth inhibition at 10 μM), 71% were intermediately toxic (higher than 50% growth inhibition at 100 μM against at least one of the cell lines), and 21% were not toxic at all (at 100 μM). Among the tested compounds, xanthones were the least toxic, while chalcones were the most toxic ones. Taken together, the primary toxicity studies revealed that scaffolds derived from aurones are the most promising candidates as they induced higher cytotoxicity among resistant cells versus sensitive cell lines (Supplementary Materials, Table S2).

Aurones [2-benzylidenebenzofuran-3(2*H*)-ones], which are structural isomers of flavones, contribute to the coloration of numerous flowers and vegetables [21]. Despite the limited number of naturally occurring aurones (compared to flavones), they are emerging as promising scaffolds in different therapeutic areas [22]. In plants, aurones are mainly hydroxylated and/or methoxylated at positions 4 and/or 6 (e.g., aureusidin, bracteatin, sulfuretin, hispidol, rengasin, and derivatives) [21]. This substitution pattern guided us to include in our primary screening the aurones bearing methoxyl groups at these positions. For the sake of optimizing aurones as cytotoxic agents, we decided to focus on azaaurones where the intracyclic oxygen of aurones was replaced with an N–H group. This modification proved to be crucial in the design of leishmanicidal and antibacterial agents [23,24,25]. Moreover, the presence of an indolinone in the core structure of azaaurones makes them closely related to natural alkaloids and pharmaceuticals exhibiting significant biological activities [26].

### 2.1. Synthesis of Azaaurones

**Scheme 1:**
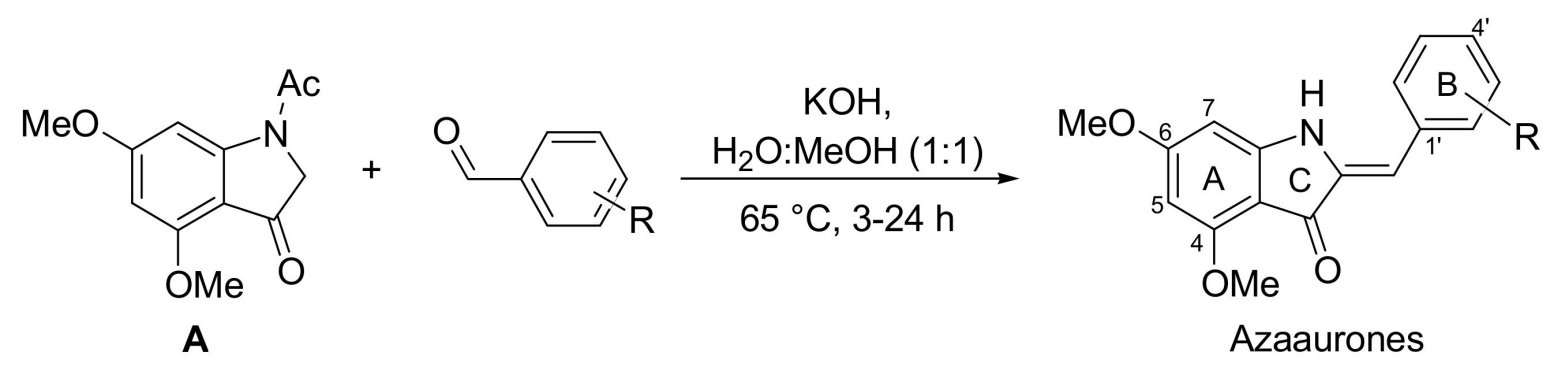
Synthesis of targeted azaaurones.

The synthesis of targeted azaaurones was carried out according to a previously reported procedure (Scheme 1). The key step was the condensation of a conveniently substituted *N*-acetylindolin-3-one **A** with a substituted benzaldehyde. It should be highlighted that *N*-deacetylation occurs during the condensation step. The indolinone moiety was prepared starting from 3,5-dimethoxyaniline and chloroacetonitrile as previously reported [23].

### 2.2. Structure–Activity Relationship Study 

We determined the cytotoxicity of the novel derivatives against the two human uterine sarcoma cell lines. Based on the IC_50_ values (in µM) determined from the effects on both cell lines, a selectivity ratio (SR) was calculated (Table 1). A value of SR > 1 implies that the compound is more cytotoxic towards P-gp-overexpressing MES-SA/Dx5 cells than the parental MES-SA cells, displaying a so-called MDR-selective activity. A value of SR < 1, on the contrary, suggests that the P-gp-expressing cells are resistant to the compound, as normally observed with P-gp substrates. In order to confirm MDR selectivity, we tested the most interesting compound (compound **4**) on further cell line pairs with or without P-gp expression and observed only a marginal selectivity (Supplementary Materials, Table S3). 

4,6-dimethoxy-4′-bromoaurone, bearing a bromine atom at the C-4′ position (on the B-ring), was one of the most active compounds identified in the primary screen, prompting the synthesis of a similar brominated azaaurone analog. Unfortunately, for unexpected reasons, this azaaurone was not stable during the purification step, whereas the chlorinated analog (compound **1**, Table 1) was obtained in its pure form. As shown in Table 1, azaaurone **1** displayed a high selectivity toward resistant cells. Next, we checked the impact of the presence of an alkyl group on the B-ring (compounds **2**–**6**) and found that butyl chains, either linear (compound **4**) or ramified (compound **6**), led to SR values higher than those obtained with ethyl and propyl groups and comparable to that of compound **1** (the SR of **4** was even slightly better), while a significant improvement in IC_50_s in both cell lines was also observed, highlighting the impact of the hydrophobicity and/or the size of the alkyl chain. On the basis of the importance of alkyl groups linked to the B-ring, we decided to investigate the presence of multiple methyl groups on this aryl moiety (azaaurones **8**–**10** and **13**). This strategy turned out to be productive as it provided the most selective compound of the series (compound **8**). However, the presence of more than two methyl groups led to less selective azaaurones (**9** and **10** versus **8** and **13**). The introduction of methoxy groups on the B-ring produced much less selective compounds as it can clearly be seen by comparing the azaaurones **8** and **11**. Taken together, the above data highlight that compounds **4** and **6**, having the lowest IC_50_s and giving SR values that were among the highest (Table 1), are the primary candidates for further development.

In order to compare the activity of azaaurones to those of well-known flavonoids highly investigated in cancer prevention and treatment, we included the naturally occurring genistein, kaempferide, and apigenin in the screens [27]. These compounds were toxic against both MES-SA (IC_50_ = 53.6 µM for genistein, 57.0 µM for kaempferide, and 36.6 µM for apigenin) and MES-SA/Dx5 cells (IC_50_ = 40.3, 39.3, and 20.3 µM for genistein, kaempferide and apigenin, respectively), as seen in Table 2. Based on these results, it can be concluded that azaaurones were more cytotoxic than these aforementioned flavonoids in most cases.

As MES-SA/Dx5 cells overexpress the efflux transporter P-gp, we checked if this transporter contributes to the selective toxicity of the most promising compounds (**4**, **5**, **6**, **7**, and **10**). The P-gp inhibitor tariquidar did not influence the selective toxicity of azaaurones, suggesting that the function of P-gp is not involved in the collateral sensitivity of MES-SA/Dx5 to these compounds (data shown in Supplementary Materials, Table S4). While multidrug-resistant MES-SA/Dx5 cells exhibit collateral sensitivity to azaaurones, in contrast to “MDR-selective” compounds (e.g., isatin thiosemicarbazones and 8-hydroxyquinoline derivatives) reported by Füredi et al. (2017) [9], our azaaurones’ selective toxicity was not influenced by the P-gp inhibitor tariquidar (TQ). These results indicate that the observed hypersensitivity of MES-SA/Dx5 cells is not linked to the activity of P-gp, and should be rather explained by off-target effects linked to other resistance mechanisms [5]. 

## 3. Materials and Methods

### 3.1. Chemistry

^1^H NMR and ^13^C NMR spectra were recorded at 200 MHz and 50 MHz, respectively, using a Bruker Avance-400 instrument. Chemical shifts (δ) are reported in ppm relative to Me_4_Si (internal standard). Electrospray ionization (ESI) mass spectra were performed by the Analytical Department of Grenoble University on an Esquire 300 Plus Bruker Daltonis instrument with a nanospray inlet. Elemental analyses were performed at the Analytical Department of Grenoble University. Thin-layer chromatography (TLC) used Merck silica gel F-254 plates (thickness 0.25 mm). Flash chromatography used Merck silica gel 60, 200–400 mesh. High-quality solvents used in chromatography were purchased from commercial sources and were used without further distillation.

### 3.2. Synthesis of Azaaurones **1**–**13**

Azaaurones were obtained by the condensation of indolinone **A** (Scheme 1) and substituted benzaldehydes [23]. Indolinone **A** was dissolved in MeOH (10 mL/mmol) and treated with KOH (50% in H_2_O, 1 mL/mmol). The solution was stirred at room temperature and allowed to reach 65 °C progressively. The reaction was quenched when TLC indicated no reaction progression (3 to 24 h). The solution was acidified by adding HCl (30 mL, 2 N), evaporated, and the crude was dissolved in H_2_O and extracted with ethyl acetate. The organic solution was separated, dried over Na_2_SO_4_, and concentrated. The crude mixture was purified by flash chromatography over silica gel, eluted with a mixture of ethyl acetate:cyclohexane (1:2) to afford pure azaaurones. The synthesized compounds were isolated in 96–98% pure form as evidenced by elemental analysis. For each azaaurone, carbon, hydrogen, and nitrogen atoms were analyzed and were found in nearly full (96–98%) agreement with the expected values. The compounds were fully characterized by ^1^H NMR, ^13^C NMR, and high-resolution mass spectrometry (HRMS) (data shown in Supplementary Materials, Table S5).

### 3.3. Biological Evaluation

In the primary screen, we investigated the toxicity of 140 compounds (dissolved in DMSO). Pipetting was performed by an automated liquid handling robot (Hamilton StarLet). Both MES-SA and MES-SA/Dx5 cells were purchased from ATCC (ATCC codes: ATCC-CRL-1976, ATCC-CRL-1977, respectively), where they were characterized by DNA fingerprinting. Upon arrival, the cells were cultured, and stocks (with passage number under 5) were frozen and stored. Cells were kept in culture for maximum 2–3 months, with passage numbers below 30. These cells were seeded for 24 h prior to drug treatment on 96-well plates (5000 cells/100 µL). Toxicity was screened in 2 concentrations (10 µM and 100 µM) for 96 h in a final volume of 200 µL. For positive control (representing toxicity), we used a toxic concentration (25 µM) of NSC-57969 [28]; and for negative control, we used 0.4% DMSO-containing medium (referring to 100% growth). Fluorescent intensity of mCherry was detected by Perkin Elmer EnSpire Multimode Plate Reader (fluorescent cells were characterized in our earlier publications) [15,29]. Excitation wavelength was set to 585 nm and emission was set to 607 nm as a result of a wavelength optimization with the instrument.

Secondary assays were performed by PrestoBlue viability reagent (Thermo Fisher Scientific). Non-fluorescent MES-SA and MES-SA/Dx5 cells were seeded on 96-well plates (5000 cells/100 µL of Dulbecco’s Modified Eagle Medium—DMEM). The next day, serially diluted compounds were added manually in an additional 100 µL volume, and plates were further incubated for 72 h. For negative control, we used 0.4% DMSO-containing DMEM; and for positive control, we prepared cell-free wells. After the 72 h incubation, the drug-containing medium was replaced by 5% PrestoBlue diluted in PBS and incubated for 1 h. Fluorescent detection of the PrestoBlue was carried out by exciting on 555 nm and reading emission on 585 nm. Tariquidar (TQ) was applied at 1 µM. The results were based on at least 3 independent experiments.

For other cell lines on which the compound **4** was tested (Supplementary Materials, Table S3): A431 and MDCK II were also purchased from ATCC; KB-3-1 and KB-V1 were provided by Dr. Michael M. Gottesman (NIH, NCI/CCR); MDCK II-B1 (which stably expresses the human wild-type ABCB1) was created by the Sleeping Beauty transposon-based gene delivery system, using the 100 × hyperactive SB transposase [30]. All cells were regularly tested for Mycoplasma contamination with the MycoAlert Mycoplasma Detection Kit from Lonza, giving negative results.

## 4. Conclusions

Targeting the collateral sensitivity of drug-resistant cancer cells is an emerging strategy developed to overcome intrinsic or acquired multidrug resistance (MDR). In this paper, we characterize novel aurone derivatives that show selective toxicity against MDR cells. Our structure–activity relationship (SAR) analysis suggests that the substitution of the intracyclic oxygen in aurones by a nitrogen atom contributes to selective toxicity. These novel azaaurone derivatives reveal a new chemotype that can serve as a starting point for further development and systematic SAR studies of a new class of MDR-selective anticancer agents.

## Figures and Tables

**Table 1:** Structures and activities of azaaurone derivatives determined from the effect on the sensitive MES-SA cell line and the resistant MES-SA/Dx5 line. IC_50_ values are in µM.

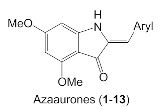				
Compound	Aryl	IC_50_ (MES-SA)	IC_50_ (Dx5)	SR ^1^
**1**	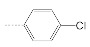	147.2 ± 32.8	36.6 ± 14.5	4.0
**2**	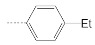	22.7 ± 1.4	10.8 ± 1.7	2.1
**3**	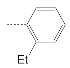	43.9 ± 3.6	13.7 ± 3.0	3.2
**4**	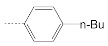	17.8 ± 4.1	3.7 ± 0.6	4.8
**5**	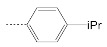	17.1 ± 1.7	5.8 ± 1.3	2.9
**6**	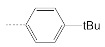	11.9 ± 1.6	3.4 ± 0.2	3.8
**7**	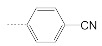	61.9 ± 10.0	13.1 ± 5.6	2.7
**8**	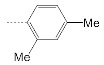	158.6 ± 43.7	32.1 ± 13.3	4.9
**9**	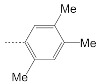	170.8 ± 4.3	131.7 ± 13.8	1.3
**10**	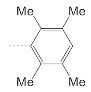	9.2 ± 2.3	19.5 ± 3.5	0.5
**11**	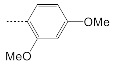	44.5 ± 12.8	27.4 ± 7.8	1.6
**12**	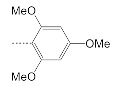	30.4 ± 2.0	15.6 ± 1.4	1.9
**13**	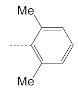	169.9 ± 30.2	44.2 ± 16.0	3.8
**Doxorubicin**		0.36 ± 0.11	4.15 ± 0.38	0.087
**NSC57969**		4.63 ± 1.31	0.59 ± 0.10	7.9

^1^ SR (selectivity ratio) is a compound’s IC_50_ against MES-SA cells (µM) divided by its IC_50_ against Dx5 cells (µM).

**Table 2:** The effect of naturally occurring flavonoids on the sensitive MES-SA cell line and the resistant MES-SA/Dx5 line. IC_50_ values are in µM.

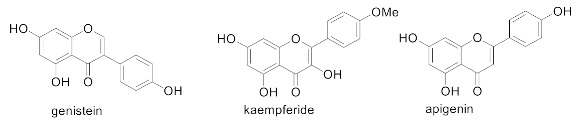			
Compound	IC_50_ (MES-SA)	IC_50_ (Dx5)	SR
Genistein	53.6 ± 4.63	40.3 ± 1.8	1.3
Kaempferide	57.0 ± 2.52	39.3 ± 3.93	1.5
Apigenin	36.6 ± 1.72	20.3 ± 1.68	1.8

## Supplementary Materials

The following are available online. Table S1: Structures of the screened compounds, Table S2: IC_50_ of aurone representatives (in µM), Table S3: Effects of azaaurone 4 in various P-gp-expressing cell lines, Table S4: Contribution of P-gp to the selective toxicity, Table S5: Physicochemical characterization of azaaurones (compounds **1**–**13**) synthesized according to Scheme 1, Figure S1: Characterization of P-glycoprotein expression and activity in MES-SA/Dx5 cells.
