# Supplementary material for: Synthesis and Anticancer Cytotoxicity of Azaaurones Overcoming Multidrug Resistance
Source: Molecules. 2020 Feb 10;25(3):764. doi: 10.3390/molecules25030764 (PMC7038029; doi:10.3390/molecules25030764)
Supplement: Supplementary file 1 [file molecules-25-00764-s001.pdf]

Supplementary Materials

Synthesis and anticancer cytotoxicity of azaaurones overcoming multidrug resistance

Szilárd Tóth [1], Áron Szepesi [1], Viet-Khoa Tran-Nguyen [2], Balázs Sarkadi [1], Katalin Német [1,4], Pierre Falson [5], Attilio Di Pietro [5,$], Gergely Szakács [1,3,$], and Ahcène Boumendjel [2,$,*]

[1] Institute of Enzymology, Research Centre for Natural Sciences, Hungarian Academy of Sciences, 1117 Budapest, Hungary;
[2] Univ. Grenoble Alpes, Département de Pharmacochimie Moléculaire (DPM), UMR 5063, 38041 Grenoble, France;
[3] Institute of Cancer Research, Medical University of Vienna, Borschkegasse 8A, A-1090 Vienna, Austria;
[4] Creative Cell Ltd., 1119 Budapest, Hungary;
[5] Institut de Biologie et Chimie des Protéines, UMR 5086, University of Lyon 1, 69007 Lyon, France.

Content

Table S1. Structures of the screened compounds. 2

Table S2. IC_{50} of aurone representatives (in μM). 6

Table S3. Effects of azaaurone 4 in various P-gp-expressing cell lines. 7

Table S4. Contribution of P-gp to the selective toxicity. 8

Table S5. Physicochemical characterization of azaaurones (compounds 1-13) synthesized according to Scheme 1. 9

Figure S1. Characterization of P-glycoprotein expression and activity in MES-SA/Dx5 cells. 11

Table S1. Structures of the screened compounds. The screened compounds are from our internal chemical libraries. The syntheses of these compounds were reported in several studies (Hadjeri, M., *J. Med. Chem.* 2**003**; Hadjeri, M., *J. Med. Chem.* **2004**; Doléans-Jordheim, A., *ChemMedChem.* **2013**; Valdameri, G., *J. Med. Chem.* **2012**; Boumendjel, A., *J. Med. Chem.* **2008**; Genoux-Bastide, *ChemMedChem.* **2012**).

4-Quinolones (20 compounds)

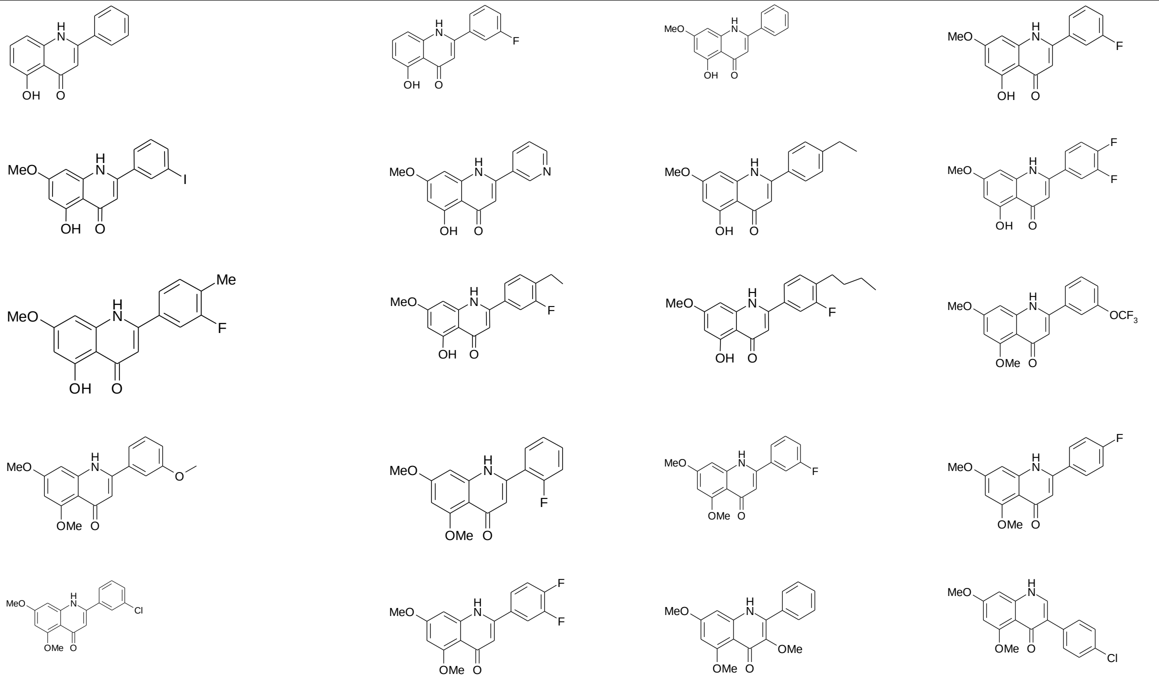

2-Quinolones (6 compounds)

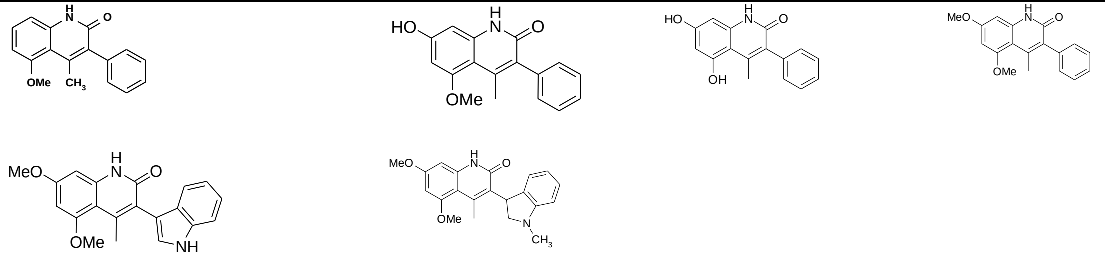

Chalcones (11 compounds)

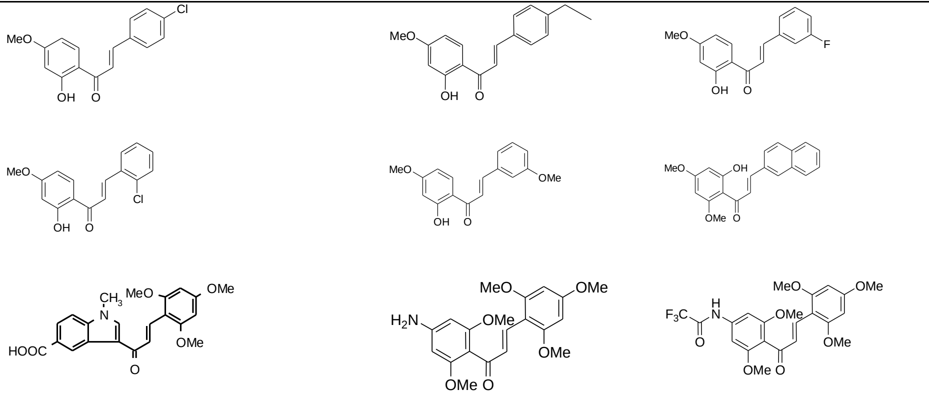

Aurones (34 compounds)

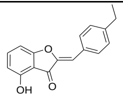 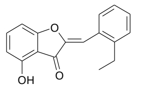 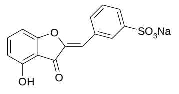 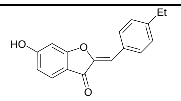

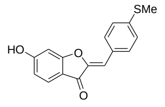 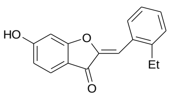 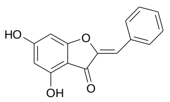 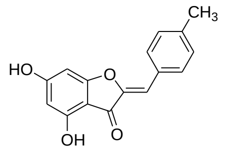

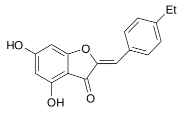 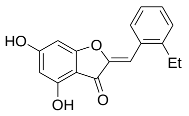 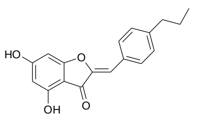 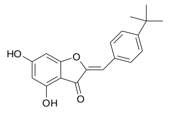

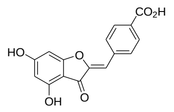 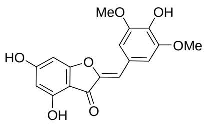 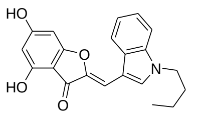 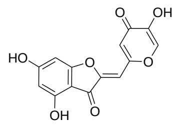

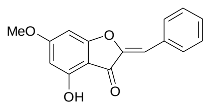 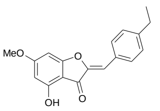 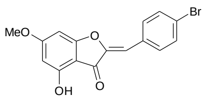 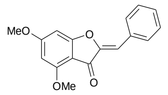

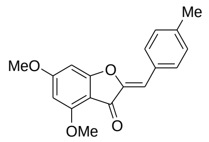 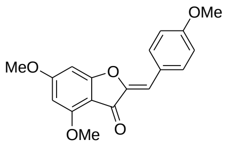 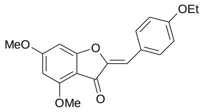 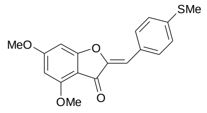

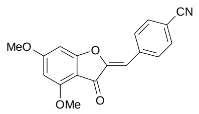 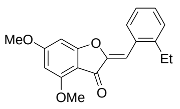 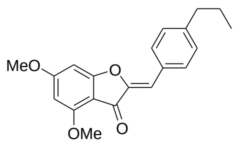 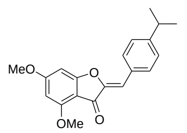

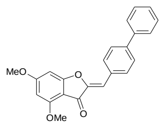 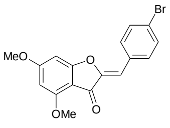 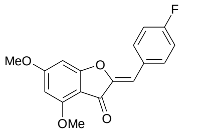 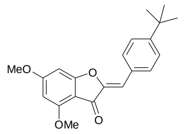

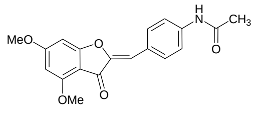 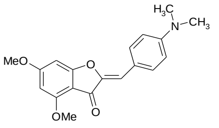

Xanthones (9 compounds)

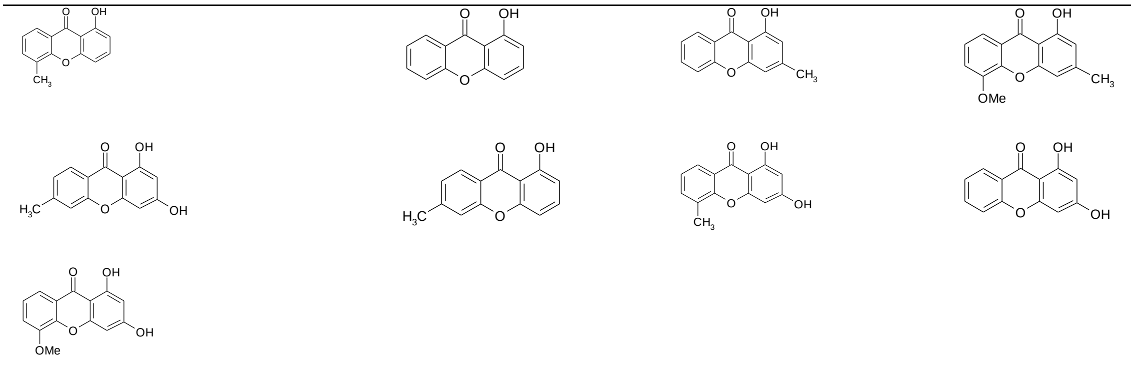

Dihydrobenzopyranes (9 compounds)

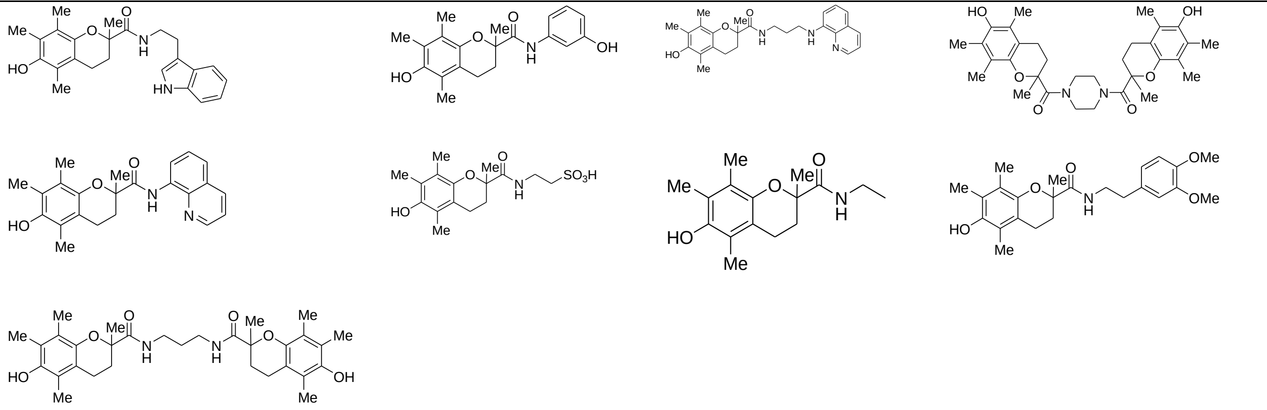

Flavones (11 compounds)

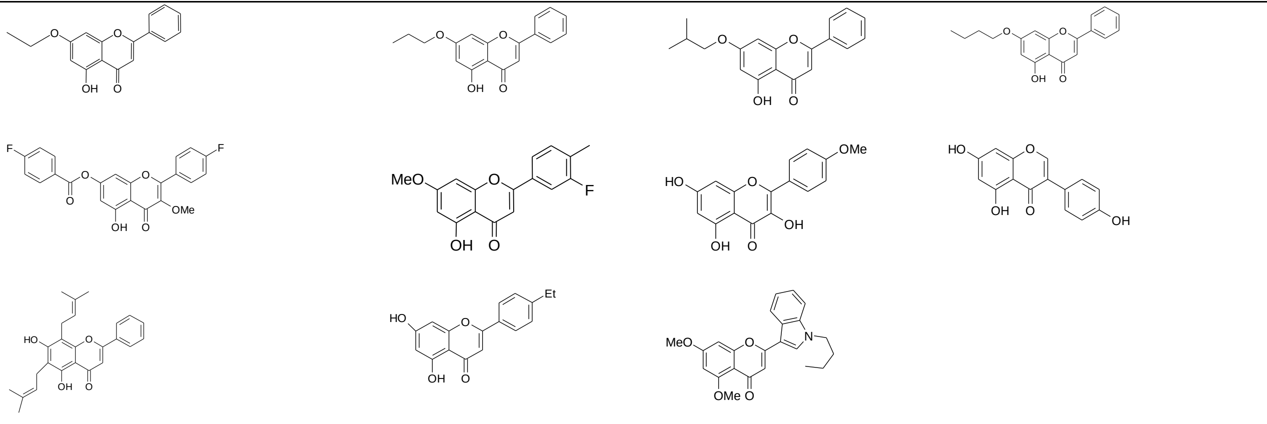

Oxadiazoles (8 compounds)

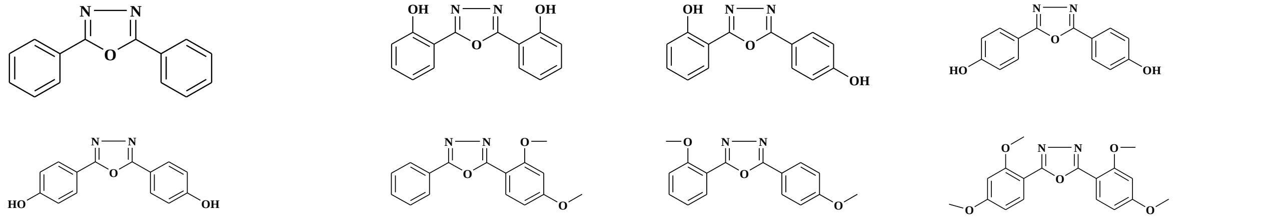

Ferulic acid derivatives (11 compounds)

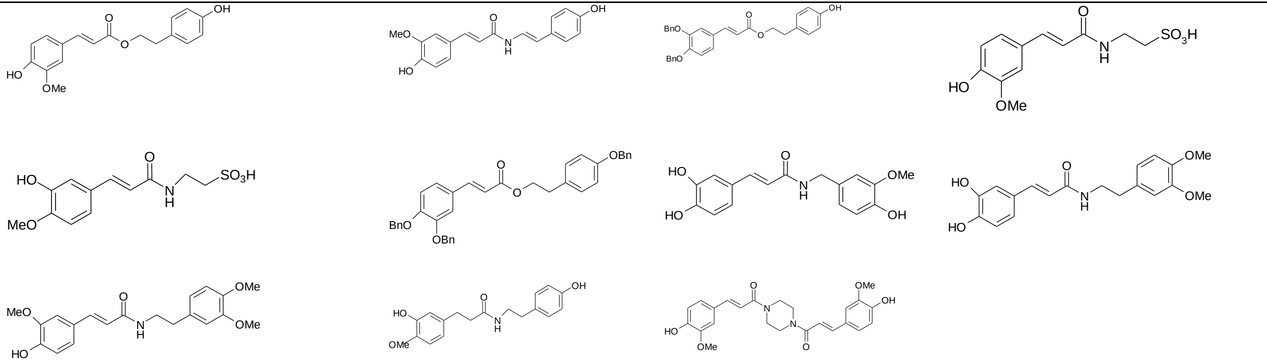

Thiosemicarbazides (15 compounds)

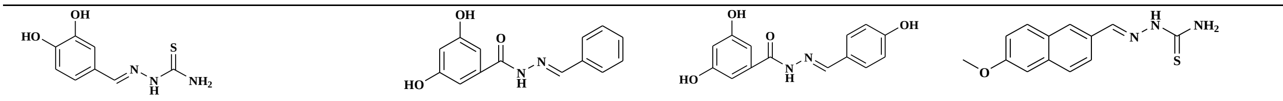

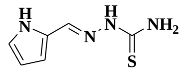
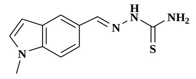
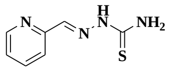
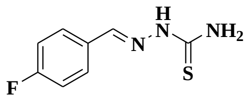
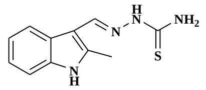
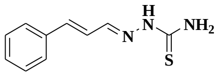
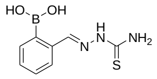
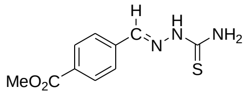
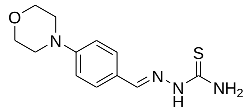
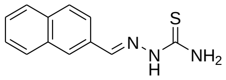
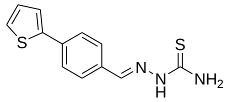

Miscellanoeus (6 compounds)

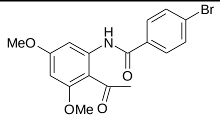
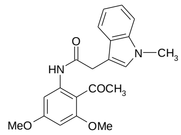
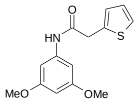
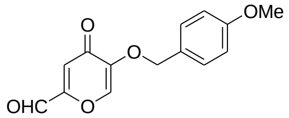
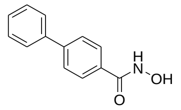
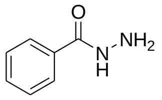

Table S2. IC_{50} of aurone representatives (in μM).

Structure	MES-SA	Dx5	MES-SA/Dx5
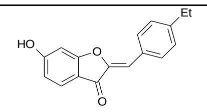	56.5 ± 5.7	43.7 ± 6.6	1.3
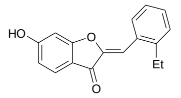	104.2 ± 20.6	91.0 ± 14.0	1.1
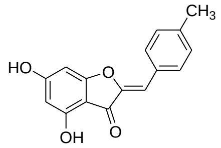	88.9 ± 15.6	56.4 ± 10.0	1.6
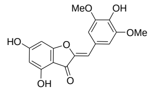	50.1 ± 10.8	109.2 ± 19.0	0.46
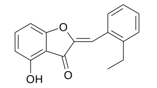	99.0 ± 18.1	53.4 ± 5.0	1.9
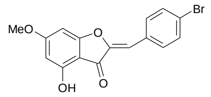	116.4 ± 24.1	44.7 ± 4.1	2.6
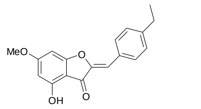	63.0 ± 1.5	32.1 ± 6.1	2.0
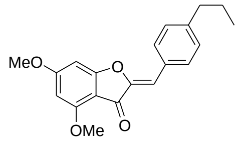	111.6 ± 30.3	38.4 ± 4.8	2.9
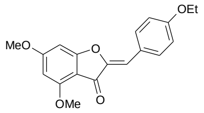	29.6 ± 1.5	21.4 ± 2.8	1.4
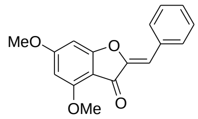	44.2 ± 0.8	18.2 ± 5.3	2.4
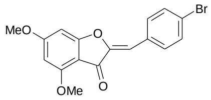	93.2 ± 6.4	33.0 ± 2.8	2.8
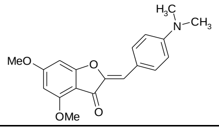	60.1 ± 3.9	15.0 ± 0.1	4.0

Table S3. Effects of azaaurone **4** in various P-gp-expressing cell lines.

**Cell lines**		**SR**
**A431**	**A431-B1**	
44.6 ± 6.9	27.0 ± 3.0	1.7
**KB-3-1**	**KB-V1**	**SR**
29.4 ± 0.4	20.9 ± 0.9	1.4
**MDCK II**	**MDCK II-B1**	**SR**
20.4 ± 4.5	16.8 ± 1.7	1.2

Values are represented as IC_{50} values ± SD (μM). SR is the abbreviation of selectivity ratio: the IC_{50} value against the parental cell line divided by the IC_{50} value against the P-gp-expressing derivative. KB-3-1 is a HeLa derived endocervical adenocarcinoma cell line. KB-V1 is its vinblastine-selected P-gp-expressing variant. MDCK II is a spontaneously immortalized canine kidney cell line. MDCK II-B1 cell line stably expressing the human wild-type ABCB1 was created by the Sleeping Beauty transposon-based gene delivery system, using the 100 × hyperactive SB transposase.

Table S4. Contribution of P-gp to the selective toxicity.

**Azaaurone**	**Mes-Sa**	**Dx5**	**SR**	**Mes-Sa(TQ)**	**Dx5(TQ)**	**SR(TQ)**
**4**	17.8 ± 4.1	3.7 ± 0.6	4.8**	3.2 ± 0.7	0.8 ± 0.2	4.0*
**5**	17.1 ± 1.7	5.8 ± 1.3	2.9*	4.3 ± 0.4	1.1 ± 0.3	3.9*
**6**	11.9 ± 1.6	3.4 ± 0.2	3.8**	2.5 ± 0.3	0.6 ± 0.01	4.2*
**7**	61.9 ± 10.0	13.1 ± 5.6	2.7**	16.9 ± 0.3	4.9 ± 0.4	3.4**
**10**	9.2 ± 2.3	19.5 ± 3.5	0.5*	9.6 ± 1.0	6.7 ± 0.7	1.4

Table S5. Physicochemical characterization of azaaurones (compounds 1-13) synthesized according to Scheme 1.

Compound	Characterization
1	**(Z)-2-(4-Chlorobenzylidene)-4,6-dimethoxy-2,3-dihydro-1*H*-indol-3-one:** Yield: 80%; yellow powder; mp 146-148 °C; **^{1}H NMR** (200 MHz, $CDCl_3$) δ 7.4 (dd, 2H, J_1 = 8.5 Hz, J_2 = 0.4 Hz, H3′, H5′), 7.3 (dd, 2H, J_1 = 8.5 Hz, J_2 = 0.4 Hz, H2′, H6′), 6.6 (s, 1H, =CH-), 6.1 (d, 1H, *J* = 1.6 Hz, H7), 5.85 (d, 1H, *J* = 1.6 Hz, H5), 3.85 (s, 3H, OCH_3), 3.8 (s, 3H, OCH_3); **^{13}C NMR** ($CDCl_3$, 50 MHz) δ 182.33, 168.89, 160.61, 156.64, 136.64, 133.87, 133.68, 130.54, 129.44, 108.36, 105.07, 91.56, 88.69, 56.06, 56.03, 27.09. **HRMS** (ESI/LTQ Orbitrap) calcd for $C_{17}H_{15}ClNO_3$ ($M+H^+$) 316.0662, found 316.0650. **Anal.** ($C_{17}H_{14}ClNO_3$) C, H, N.
2	**(Z)-2-(4-Ethylbenzylidene)-4,6-dimethoxy-2,3-dihydro-1*H*-indol-3-one:** Yield: 70%; yellow powder; mp 215-217 °C; **^{1}H NMR** (200 MHz, $CDCl_3$) δ 7.45 (dd, 2H, J_1 = 8.2 Hz, J_2 = 0.4 Hz, H2′, H6′), 7.25 (dd, 2H, J_1 = 8.2 Hz, J_2 = 0.4 Hz, H3′, H5′), 7.1 (bs, 1H), 6.7 (s, 1H), 6.1 (d, 1H, *J* = 1.8 Hz, H7), 5.9 (d, *J* = 1.8 Hz, 1H, H5), 3.9 (s, 3H), 3.8 (s, 3H), 2.7 (q, 2H, *J* = 6 Hz, $-CH_2$), 1.3 (t, *J* = 6 Hz, 3H, CH_3); **^{13}C NMR** (CD_3COCD_3, 50 MHz) δ 182.89, 169.88, 161.74, 158.71, 145.59, 137.21, 134.12, 130.91, 129.89, 108.70, 105.62, 92.23, 90.13, 56.77, 56.60, 16.56. **HRMS** (ESI/LTQ Orbitrap) calcd for $C_{19}H_{20}NO_3$ $[M+H]^+$. 310.1365, found 310.1365. **Anal.** ($C_{19}H_{19}NO_3$) C, H, N.
3	**(Z)-2-(2-Ethylbenzylidene)-4,6-dimethoxy-2,3-dihydro-1*H*-indol-3-one:** Yield: 74%; yellow powder; mp 197-198 °C; **^{1}H NMR** (400MHz, $CDCl_3$) δ 7.51-7.49 (m, H6), 7.25 (bs, 3H, H3′, H4′, H5′), 6.90 (s, 1H, =CH-), 6.78 (bs, 1H, NH), 6.00 (s, 1H, H7), 5.92 (s, 1H, H5), 3.94 (s, 3H, OCH_3), 2.79-2.73 (q, *J* = 7.28 Hz, 2H, CH_2), 1.22-1.18 (t, *J* = 7.52 Hz, 3H, CH_3); **^{13}C NMR** ($CDCl_3$, 100 MHz) δ 183.58, 170.14, 162.06, 158.04, 145.81, 138.58, 134.67, 130.76, 129.95, 129.83, 127.77, 109.11, 106.66, 92.53, 89.58, 57.38, 57.34, 28.22, 16.04. **HRMS** (ESI/LTQ Orbitrap) calcd for $C_{19}H_{20}NO_3$ $[M+H]^+$. 310.1028, found 310.1109. **Anal.** ($C_{19}H_{19}NO_3$) C, H, N.
4	**(Z)-2-(4-Buthylbenzylidene)-4,6-dimethoxy-2,3-dihydro-1*H*-indol-3-one:** Yield: 59%; yellow powder; mp 90-92 °C; **^{1}H NMR** (400 MHz, $CDCl_3$) δ 7.44 (d, *J* = 8.2 Hz, 2H, H2′, H6′), 7.27 (d, *J* = 8.2 Hz, 2H, H3′, H5′), 6.8 (bs, 1H, NH), 6.75 (s, 1H, =CH-), 6.07 (d, *J* = 1.69 Hz, 1H, H7), 5.93 (d, *J* = 1.72 Hz, 1H, H5), 3.92 (s, 3H, OCH_3), 3.87 (s, 3H, OCH_3), 2.63 (t, *J* = 7.5 Hz, 2H, $\underline{CH_2}$-Ph), 1.63 (bs, 4H, CH_2-CH_2), 0.94 (t, *J* = 7.5 Hz, 3H, CH_3); **^{13}C NMR** ($CDCl_3$, 100 MHz) δ 170.02, 161.91, 158.5, 144.86, 137.37, 133.89, 130.85, 111.56, 106.62, 92.69, 89.85, 57.33, 37.06, 35.01, 90.3, 23.91, 15.51. **HRMS** calcd for $C_{21}H_{23}NO_3$: 337.42, found 338.22 $[M+H]^+$. **Anal.** ($C_{21}H_{23}NO_3$) C, H, N.
5	**(Z)-2-(4-Isopropylbenzylidene)-4,6-dimethoxy-2,3-dihydro-1*H*-indol-3-one:** Yield: 53%; yellow powder; mp 252 °C; **^{1}H NMR** (400 MHz, $CDCl_3$) δ 7.47 (d, 2H, *J* = 4 Hz, H2′, H6′), 7.31 (d, 1H, *J* = 4 Hz, H3′, H5′), 6.86 (bs, 1H, NH), 6.77 (s, 1H, =CH-), 6.07 (d, 1H, *J* = 1.6 Hz, H5), 5.94 (d, 1H, *J* = 2 Hz, H7), 3.94 (s, 3H, OCH_3), 3.88 (s, 3H, OCH_3), 3.01 (m, 1H, $\underline{CH}(CH_3)_2$), 1.28 (d, 6H, *J* = 6.8 Hz, $(CH_3)_2$); **^{13}C NMR** ($CDCl_3$, 100 MHz) δ 179.94, 166.27, 158.10, 154.23, 147.01, 133.50, 130.23, 127.22, 124.97, 107.99, 102.78, 88.95, 86.07, 53.88, 53.62, 31.69, 21.58, 21.48. **HRMS** calcd for $C_{20}H_{21}NO_3$: 323.5857, found 323.6541. **Anal.** ($C_{20}H_{21}NO_3$) C, H, N.
6	**(Z)-2-(4-tert-Buthylbenzylidene)-4,6-dimethoxy-2,3-dihydro-1*H*-indol-3-one:** Yield: 51%; brown powder; mp 196-197 °C; **^{1}H NMR** ($CDCl_3$, 400 MHz) δ 7.46 (bs, 2H, H2′, H6′), 7.25 (bs, 2H, H3′, H5′), 6.84 (bs, 1H, NH), 6.77 (s, 1H, =CH-), 6.06 (d, *J* = 1.69 Hz, 1H, H7), 5.94 (d, *J* = 1.67 Hz, 1H, H5), 3.94 (s, 3H, OCH_3), 3.87 (s, 3H, OCH_3), 1.37 (bs, 9H, $-C(CH_3)_3$); **^{13}C NMR** ($CDCl_3$, 400 MHz) δ 184.07, 170.04, 161.92, 158.12, 152.94, 137.50, 133.69, 130.70, 127.57, 111.34, 106.59, 92.67, 89.88, 57.32, 36.30, 32.73. **HRMS** calcd for $C_{21}H_{23}NO_3$: 337.42, found 336.22 $[M-H]^+$. **Anal.** ($C_{21}H_{23}NO_3$) C, H, N.
7	**(Z)-2-(4-Cyanobenzylidene)-4,6-dimethoxy-2,3-dihydro-1*H*-indol-3-one:** Yield: 82%; yellow powder; mp 135 °C; **^{1}H NMR** (400 MHz, $CDCl_3$) δ 7.46 (d, 2H, *J* = 8.4 Hz, H3′, H5′), 7.39 (d, 2H, *J* = 8.4 Hz, H2′, H6′), 6.80 (bs, 1H, NH), 6.62 (s, 1H, =CH-), 6.01 (d, 1H, *J* = 2 Hz, H7), 5.88 (d, 1H, *J* = 1.6 Hz, H5), 3.85 (s, 3H, OCH_3), 3.81 (s, 3H, OCH_3), 3.11 (s, 1H, CH); **^{13}C NMR** ($CDCl_3$, 100 MHz) δ 182.19, 168.75, 140.47, 156.50, 136.50, 133.73, 133.54, 130.40, 129.31, 108.21, 104.93, 91.52, 88.55, 77.27, 55.92, 55.88, 26.95. **SM** (EI): m/z 305 $[M]^+$. **HRMS** (ESI/LTQ Orbitrap) calcd for $C_{18}H_{14}N_2O_3$ 306.1004, found 307.0990 $[M+H]^+$. **Anal.** ($C_{18}H_{14}N_2O_3$) C, H, N.

**8**	**(Z)-2-(2,4-Dimethylbenzylidene)-4,6-dimethoxy-2,3-dihydro-1*H*-indol-3-one:** Yield: 45%; brown powder; mp 235 °C; **^{1}H NMR** (200 MHz, CD_3COCD_3) δ 7.5 (m, 1H, H2′), 7.06 (m, 2H, H4′, H6′), 6.6 (s, 1H, =CH-), 6.2 (d, *J* = 1.8 Hz, 1H, H7), 5.99 (d, *J* = 1.78 Hz, 1H, H5), 3.67 (s, 3H, OCH_3), 3.65 (s, 3H, OCH_3), 2.34 (s, 3H, CH_3), 2.28 (s, 3H, CH_3); **^{13}C NMR** (CD_3COCD_3, 50 MHz) δ 182.07, 169.26, 161.19, 158.18, 138.42, 138.36, 137.22, 132.19, 131.81, 129.26, 127.79, 105.56, 105.11, 91.47, 89.39, 56.18, 56.02, 21.28, 20.13. **HRMS** calcd for $C_{19}H_{19}NO_3$: 309.37, found 310.23 $[M+H]^+$. **Anal.** ($C_{19}H_{19}NO_3$) C, H, N.
**9**	**(Z)-2-(2,4,5-Trimethylbenzylidene)-4,6-dimethoxy-2,3-dihydro-1*H*-indol-3-one:** Yield: 10%; yellow powder; mp 229 °C; **^{1}H NMR** (400 MHz, $CDCl_3$) δ 7.27 (s, 1H, H6′), 7.03 (s, 1H, H3′) 6.84 (bs, 2H, NH, =CH-), 6.04-6.03 (d, *J* = 1.61 Hz, 1H, H7), 5.91 (d, *J* = 1.58 Hz, 1H, H5), 3.92 (s, 3H, OCH_3), 3.86 (s, 3H, OCH_3), 2.33 (s, 3H, CH_3), 2.28 (s, 3H, CH_3), 2.25 (s, 3H, CH_3); **^{13}C NMR** ($CDCl_3$, 100 MHz) δ 183.56, 169.95, 162.03, 157.89, 138.46, 137.83, 132.29, 135.87, 13.86, 132.73, 130.62, 109.71, 106.84, 92.56, 89.55, 57.41-57.34, 21.13. **HRMS** calcd for $C_{20}H_{21}NO_3$: 323.40, found 324.19 $[M+H]^+$. **Anal.** ($C_{20}H_{21}NO_3$) C, H, N.
**10**	**(Z)-2-(2,3,5,6-Tetramethylbenzylidene)-4,6-dimethoxy-2,3-dihydro-1*H*-indol-3-one:** Yield: 18%; yellow powder; mp 260 °C; **^{1}H NMR** (400 MHz, $CDCl_3$) δ 6.97 (s, 1H, H4′), 6.85 (s, 1H, =CH-), 5.89 (d, *J* = 1.66 Hz, 1H, H7), 5.87 (d, *J* = 1.69 Hz, 1H, H5), 3.93 (s, 3H, OCH_3), 3.82 (s, 3H, OCH_3), 2.25 (s, 6H, 2 CH_3), 2.15 (s, 6H, 2 CH_3); **^{13}C NMR** ($CDCl_3$, 100 MHz) δ 182.79, 170.18, 162.10, 157.57, 139.32, 135.63, 134.55, 134.02, 132.65, 111.82, 92.24, 89.08, 57.43, 57.29, 21.64, 18.33. **HRMS** calcd for $C_{21}H_{23}O_3N$: 337.41, found 338.23 $[M+H]^+$. **Anal.** ($C_{21}H_{23}NO_3$) C, H, N.
**11**	**(Z)-2-(2,4-Dimethoxybenzylidene)-4,6-dimethoxy-2,3-dihydro-1*H*-indol-3-one:** Yield: 46%; orange powder; mp 214-216 °C; **^{1}H NMR** (400 MHz, $CDCl_3$) δ 7.42-7.39 (d, 1H, *J* = 8.42, H6′), 6.82 (s, 1H, H3′), 6.56 (d, 1H, *J* = 8.37 Hz, H5′), 6.51 (s, 1H, =CH-), 6.01 (s, 1H, H7), 5.89 (s, H, H5), 3.92 (s, 6H, 2 OCH_3), 3.86 (s, 3H, OCH_3), 3.85 (s, 3H, OCH_3); **^{13}C NMR** ($CDCl_3$, 100 MHz) δ 183.56, 169.95, 162.03, 157.89, 138.46, 137.83, 132.29, 135.87, 13.86, 132.73, 130.62, 109.71, 106.84, 92.56, 89.55, 57.41, 57.34, 21.13. **HRMS** calcd for $C_{19}H_{19}NO_5$: 341,369, found 342.42 $[M+H]^+$. **Anal.** ($C_{19}H_{19}NO_5$) C, H, N.
**12**	**(Z)-2-(2,4,6-Trimethoxybenzylidene)-4,6-dimethoxy-2,3-dihydro-1*H*-indol-3-one:** Yield: 60%; yellow powder; mp 109-111 °C; **^{1}H NMR** (400 MHz, $CDCl_3$) δ 7.29 (bs, 1H, NH), 6.98 (s, 1H, =CH-), 6.19 (bs, 2H, H3′, H5′), 5.97 (d, 1H, *J* = 1.51 Hz, H7), 5.86 (d, 1H, *J* = 1.47 Hz, 1H, H5), 3.91 (s, 3H, OCH_3), 3.89 (s, 6H, 2 OCH_3), 3.85 (s, 6H, 2 OCH_3); **^{13}C NMR** ($CDCl_3$, 100 MHz) δ 182.34, 168.20, 162.09, 160.48, 159.32, 155.44, 135.60, 106.88, 105.31, 102.19, 92.04, 90.52, 87.86, 56.61, 56.00, 55.83, 55.64. **HRMS** calcd $C_{20}H_{21}NO_6$: 371.396, found 372.06 $[M+H]^+$. **Anal.** ($C_{20}H_{21}NO_6$) C, H, N.
**13**	**(Z)-2-(2,6-Dimethylbenzylidene)-4,6-dimethoxy-2,3-dihydro-1*H*-indol-3-one:** Yield: 25%; yellow powder; mp 217-218 °C; **^{1}H NMR** (400 MHz, $CDCl_3$) δ 7.31-7.03 (m, *J* = 7.62 Hz, 4H, H3′, H4′, H5′, NH), 6.84 (s, 1H, =CH-), 6.03 (s, 1H, H7), 5.92 (s, 1H, H5), 3.93 (s, 3H, OCH_3), 3.86 (s, 3H, OCH_3), 2.37 (s, 3H, CH_3), 2.35 (s, 3H, CH_3); **^{13}C NMR** ($CDCl_3$, 100 MHz) δ 183.49, 170.05, 162.09, 157.90, 138.21, 137.24, 136.81, 135.27, 132.35, 130.49, 130.05, 109.52, 106.79, 92.53, 89.56, 57.47, 57.35, 22.69, 21.19. **HRMS** calcd for $C_{19}H_{19}NO_3$: 309.37, found 310.23 $[M+H]^+$. **Anal.** ($C_{19}H_{19}NO_3$) C, H, N.

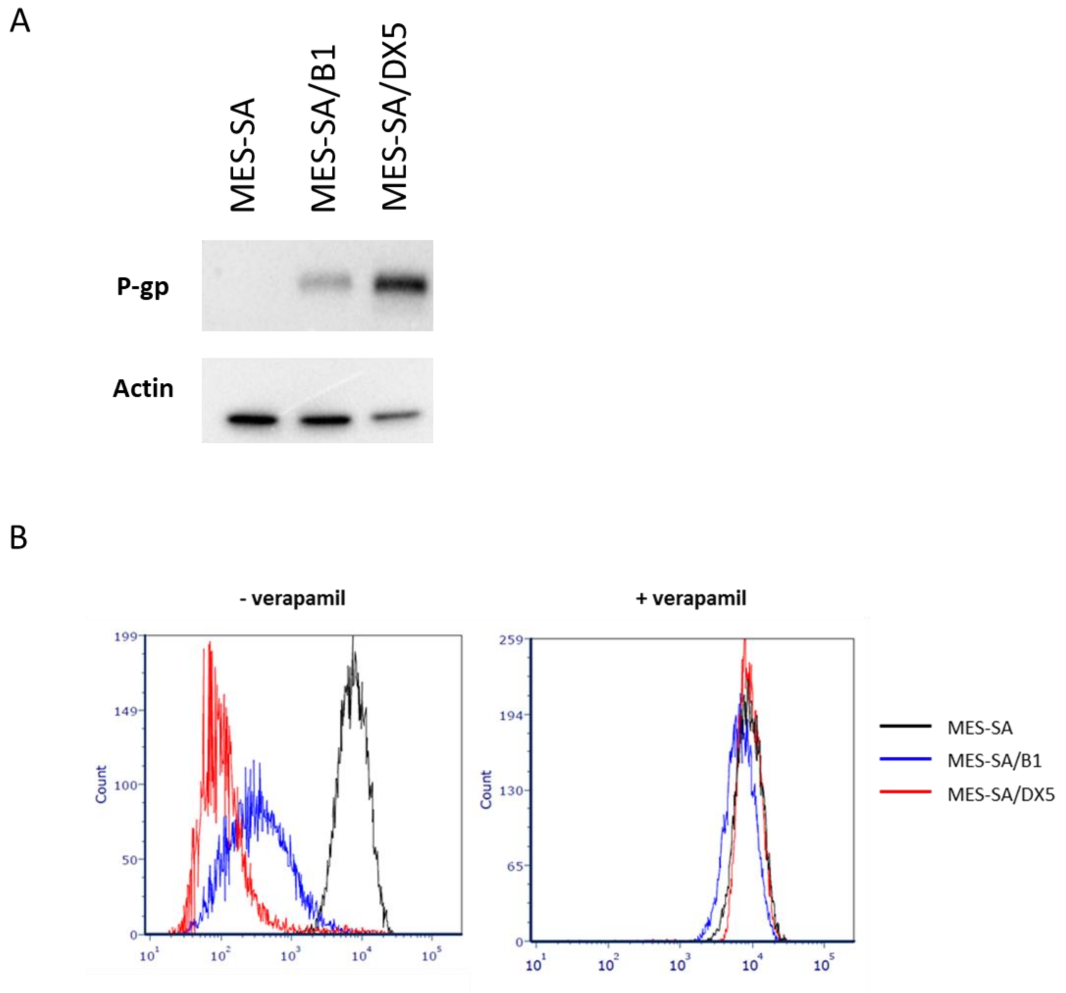

Figure S1. Characterization of P-glycoprotein expression and activity in MES-SA/Dx5 cells. **(A)** P-gp (upper lane) was visualized by Western blotting using the C219 antibody; Beta-actin is shown as loading control (lower lane). **(B)** Characterization of P-gp activity with the calcein assay. Calcein-AM is extruded by P-gp from MES-SA/Dx5 cell lines, preventing the accumulation of fluorescent calcein in the cells (left). Inhibition of the transporter by verapamil (10 μM) restores fluorescence to the levels observed in parental MES-SA cells (right). MES-SA/B1 cells, expressing P-gp by viral transduction, are shown as control (Cserepes *et al.*, 2019).
